# Supplementary material for: Novel Flavin Mononucleotide-Functionalized Cerium Fluoride Nanoparticles for Selective Enhanced X-Ray-Induced Photodynamic Therapy
Source: J Funct Biomater. 2024 Dec 10;15(12):373. doi: 10.3390/jfb15120373 (PMC11676406; doi:10.3390/jfb15120373)

Supplementary materials

Table S1. Characteristics of NPs (hydrodynamic diameter and ζ-potential)

Sample	Hydrodynamic diameter in deionized water, nm	PDI	ζ-potential in deionized water, mV
CeF_3	66±9	0. 314±0.021	+37.48
CeF_3-FMN	74±5	0.244±0.016	+40.59

Table S2. Values of absorption, integral intensity and quantum yield of fluorescence of NPs

	Absorbance	Integrated fluorescence intensity	Quantum yield
**Tyrosine (reference)**	0.0267<br>0.0534<br>0.104	3123.4<br>6137.0<br>11259.6	0.13±0.01
**CeF_3**	0.0135<br>0.0254<br>0.0379	4379.3<br>8543.6<br>13588.9	0.42 ± 0.02
**CeF_3+FMN**	0.0260<br>0.0506<br>0.0983	2628.0<br>4945.8<br>9392.8	0.11 ± 0.02

Table S3. Fitting results of the L929 and A431 cells dose-response curves. The 37% survival dose (D0) is obtained by interpolation from the fit curves.

NPs	Concentration, M	D_0, Gy	α	β	α/β
**L929**					
Control		2.7	0.07486	0.10910	0.6863
CeF_3	10^{-7}	4.5	-0.06921	0.06432	-1.0760
	10^{-3}	4.8	0.18170	0.00524	34.6800
CeF_3+FMN	10^{-7}	6.9	0.02610	0.01565	1.6680
	10^{-3}	5.3	0.12390	0.01168	10.6000
**A431**					
Control		3.5	0.28900	-0.00094	-308.2000
CeF_3	10^{-7}	2.5	0.24470	0.05531	4.4240
	10^{-3}	1.8	0.58130	-0.02395	-24.2700
CeF_3+FMN	10^{-7}	2.5	0.30710	0.03446	8.9110
	10^{-3}	2.2	0.42980	0.01412	30.4400

Figure S1. The STEM image of CeF_3 NPs

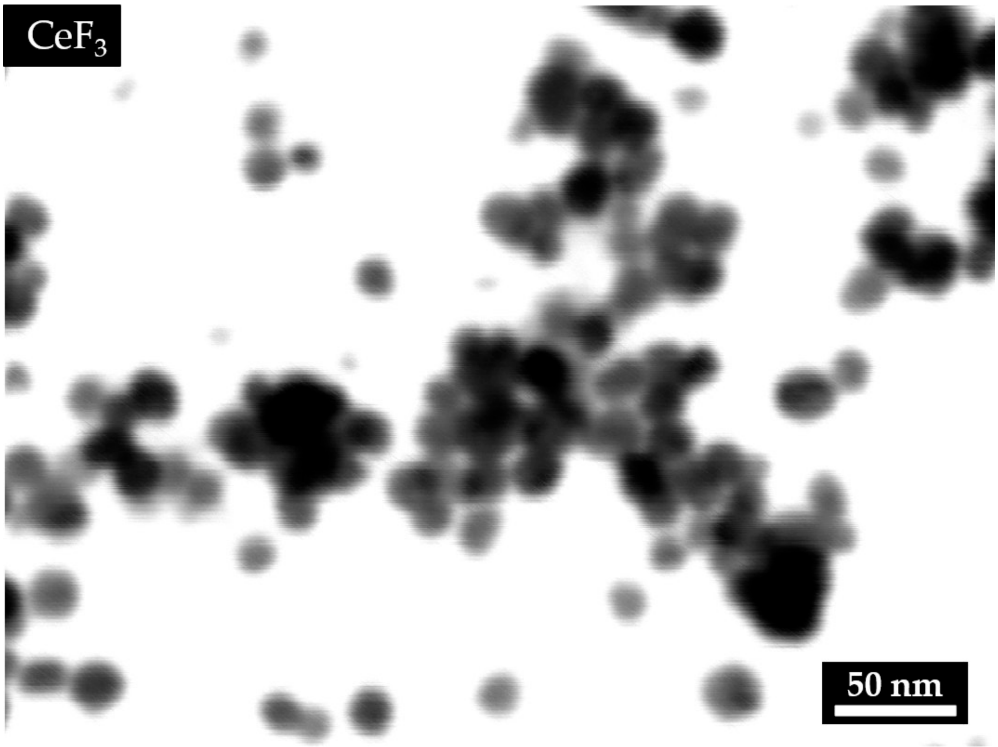

Figure S2. EDX spectrum of CeF_3 (a) and CeF_3-FMN (b) NPs

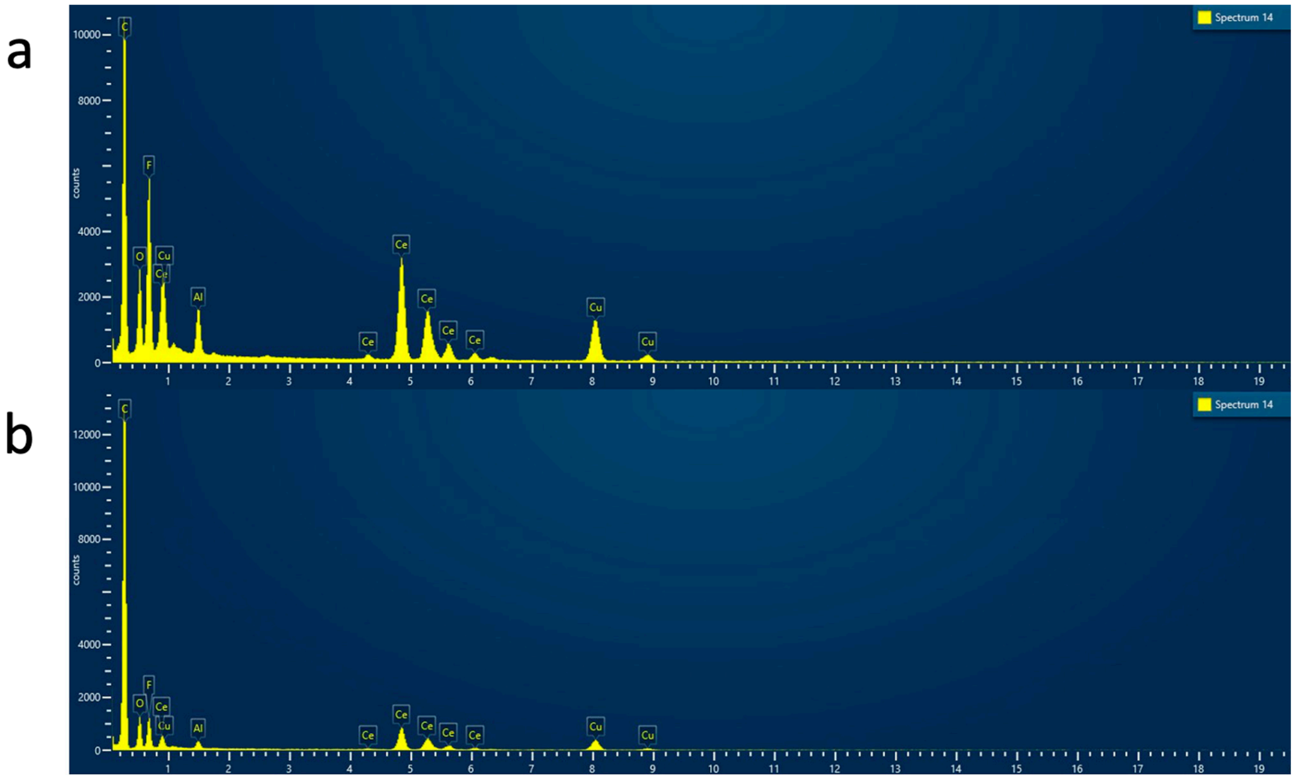

Supplement: Supplementary file 1 [file jfb-15-00373-s001.zip › jfb-3330779-supplementary.pdf]
